# 

**Published:** 1881-03-20

**Authors:** 


### *AMERICAN MEDICAL ASSOCIATION.*

The Thirty-Second Annual Session will be held in Richmond, Va., on Tuesday, Wednesday, Thursday, and Friday May 3, 4, 5, 6, 1881, commencing on Tuesday at 11 A. M.

"The delegates shall receive their appointment from permanently organized State Medical Societies, and such County and District Medical Societies as are recognized by *representation in their respective State Societies*, and from the Medical Department of the Army and Navy, and the Marine Hospital Service of the United States."

"Each State, County, and District Medical Society entitled to representation shall have the privilege of sending to the Association one delegate for every ten of its regular resident members, and one for every additional fraction of more than half that number; *Provided*, however, that the number of delegates for any particular State, territory, county, city, or town shall not exceed the ratio of one in ten of the resident physicians who may have signed the Code of Ethics of the Association."

☞Secretaries of Medical Societies as above designated are earnestly requested to forward, *at once*, lists of their delegates.

*Sections*.—"The chairman of the several Sections shall prepare and read in the general sessions of the Association, papers on the advances and discoveries of the past year in the branches of science included in their resp ctive Sections. . . . ."—BY-LAWS, Art. II., Sect. 4.

Practice of Medicine, Materia Medica, and Physiology—Dr. Wm. Pepper, 1811 Spruce St., Phila., Pa., Chairman; Dr. T. A. Ashby, Baltimore, Md., Secretary.

Obstetrics and Diseases of Women and Children—Dr. Jas. R. Chadwick, cor. Marlborough and Clarendon Sts., Boston, Mass., Chairman; Dr. Jos. Taber Johnson, Washington, D. C., Secretary.

Surgery and Anatomy—Dr. Hunter McGuire, Richmond, Va., Chairman; Dr. Duncan Eve, Nashville, Tenn., Secretary.

State Medicine—Dr. Jas. T. Reeve, Appleton, Wis., Chairman; Dr. R. G. Jennings, Little Rock, Ark., Secretary.

Ophthalmology, Otology, and Laryngology—Dr. Dudley S. Reynolds, Louisville, Ky., Chairman; Dr. Swan M. Burnett, Washington, D. C., Secretary.

Diseases of Children—Dr. A. Jacobi, 110 W. 34th St., New York, Chairman; Dr. T. M. Rotch, 77 Marlborough St., Boston, Mass., Secretary.

☞The member desiring to read a paper before any Section should forward the paper, or its *title* and *length*, (not to exceed twenty minutes in reading) to the Chairman of the Committee of Arrangements at least one month before the meeting.—*By-Laws.*

*Committee of Arrangements*.—Dr. F. D. Cunningham, Richmond, Va., Chairman.

*Amendment to the By-Laws* offered by Dr. J. M. Keller, Ark.: In the election of Officers and the appointment of Committees by this Association and its President, they shall be confined to members and delegates present at the meeting, except in the Committees of Arrangements, Climatology, and Credentials.

W. H. ATKINSON, Permanent Secretary.

### *RECEIPTED.*

[Receipts not acknowledged privately are entered here.]

1880—Drs. R A McIntosh, B H Mathews, T W Spruell.

1881—Drs. J R Wilson. W H Pool, D S Aswell, T L Doster, J H Blain, E A Anderson, J F Pou, W F Jacobs, M H [illegible]ys, F R Van Eaton, J M Jackson, R J Tolbert, J H Jennings, E Wheeler, T L Quilli[illegible]n, P P Terry, Joseph Underwood, B M Walker, Jno R Mason, W F Clary, J D Jordan, J S Milling, T F Babbitt, T S Parrham, J W Gilbert, A Harris, J M Stansell, C P Sanders, W R Brawner, B T Phipps, W S Glass, H Allison, J B Rumph, J O Sanders, J R Johnson, W P Chapman, Louis Hadden.

